# Overexpression of MET4 Leads to the Upregulation of Stress-Related Genes and Enhanced Sulfite Tolerance in *Saccharomyces uvarum*

**DOI:** 10.3390/cells11040636

**Published:** 2022-02-11

**Authors:** Zhuo Wei, Zhiming Zhang, Wenjuan Zhao, Tuo Yin, Xiaozhen Liu, Hanyao Zhang

**Affiliations:** 1 Key Laboratory for Forest Resources Conservation and Utilization in the Southwest Mountains of China, Ministry of Education, Southwest Forestry University, Kunming 650224, China; wz@swfu.edu.cn (Z.W.); zhangzhiming@swfu.edu.cn (Z.Z.); zhao@swfu.edu.cn (W.Z.); 2 Key Laboratory of Biodiversity Conservation in Southwest China, National Forest and Glassland Administration, Southwest Forestry University, Kunming 650224, China; yintuo@swfu.edu.cn

**Keywords:** *Saccharomyces uvarum*, *MET4*, gene function, fermentation weight loss analysis, transcriptome analysis, RT-qPCR

## Abstract

*Saccharomyces uvarum* is one of the few fermentative species that can be used in winemaking, but its weak sulfite tolerance is the main reason for its further use. Previous studies have shown that the expression of the methionine synthase gene (*MET4*) is upregulated in *FZF1* (a gene encoding a putative zinc finger protein, which is a positive regulator of the transcription of the cytosolic sulfotransferase gene *SSU1*) overexpression transformant strains, but its exact function is unknown. To gain insight into the function of the *MET4* gene, in this study, a *MET4* overexpression vector was constructed and transformed into *S. uvarum* strain A9. The *MET4* transformants showed a 20 mM increase in sulfite tolerance compared to the starting strain. Ninety-two differential genes were found in the transcriptome of A9-*MET4* compared to the A9 strain, of which 90 were upregulated, and two were downregulated. The results of RT-qPCR analyses confirmed that the expression of the HOMoserine requiring gene (*HOM3*) in the sulfate assimilation pathway and some fermentation-stress-related genes were upregulated in the transformants. The overexpression of the *MET4* gene resulted in a significant increase in sulfite tolerance, the upregulation of fermentation-stress-related gene expression, and significant changes in the transcriptome profile of the *S. uvarum* strain.

## 1. Introduction

The quality of wine depends, to a certain extent, on the species or strain of yeast used in the fermentation process [1]. In addition to the common brewing yeasts, *Saccharomyces uvarum* is often used in winemaking because of its ability to ferment at low temperatures and its ability to produce specific aromas [2]. Sulfites are known to be a widely used preservative that is toxic to many microorganisms, and can also give wines a specific flavor during fermentation in the presence of yeast [3]. There are no compounds that can completely replace this additive because of its multifunctional properties, for example, the inhibition of the growth and enzymatic activity of the other microorganisms during winemaking and preservation [3,4]. However, sulfites not only destroy the cell structure, but also bind to some enzymes or metabolites, hindering the normal metabolic activities of the cells and seriously affecting the fermentation efficiency of the winemaking yeast in the later stages of fermentation [5]. Therefore, sulfite resistance in winemaking yeast is considered to be a vital trait in winemaking.

An overexpression analysis, transcriptome analysis, and quantitative reverse transcription-polymerase chain reaction (RT-qPCR) analysis are effective methods for gene function analysis and have been used in many applications in the study of gene function in *S. cerevisiae* [6,7] and *S. uvarum* [8,9]. In sulfite tolerance studies, the sulfotransferase gene (*SSU1*) was first identified as an important gene in the regulation of sulfite tolerance traits, and its overexpression significantly enhanced sulfite tolerance in *S. cerevisiae* [10]. *S. cerevisiae* cells have different mechanisms to deal with the stress of sulfite production, including an increased production of bound acetaldehyde, regulation of the sulfite uptake pathway, and efflux of sulfite through a plasma membrane pump encoded by the *SSU1* gene [11]. Another important gene for sulfite resistance is the five zinc fingers protein encoding gene (*FZF1*), a positive regulator of *SSU1* gene transcription. The *FZF1* gene encodes a 5-finger transcription factor that plays a vital role in sulfite resistance in *S. cerevisiae*. The protein encoded by *FZF1* in *S. cerevisiae* contains five C2H2-type zinc finger structural domains, whereas in *S. uvarum*, only four of the proteins encoded by the *FZF1* gene are present [12,13]. Sulfite resistances of many *S. cerevisiae* strains are conferred by changes in *FZF1* expression and changes in protein structure.

*MET4*p, encoded by the methionine synthase gene (*MET4*), is a transcriptional activator belonging to the family of leucine zip proteins [12]. *MET4*p can stimulate the positive transactivator of *MET* gene transcription in the methionine biosynthetic pathway [14], participating in the transcriptional activation of *Met28* [15] and *Met30* [16], and resisting sulfur stress by assembling the *MET4*–*Met28*–*MET*31 [17] and *MET4*–*Met28*–*Met32* [18] complexes, inducing glutathione synthesis [18], and so on. Thus, *MET4* can play a vital regulatory role in the relative stability of *Saccharomyces cerevisiae* in response to sulfur stress.

There are just two reports on the mechanism of sulfite tolerance in *S. uvarum* [8,9]. Our previous study found that the sulfite tolerance-related gene *FZF1* regulates the expression of *MET4* and *HAL4*, but not *SSU1* [9]. Meanwhile, elevating the expression of the *SSU1* gene could also enhance the sulfite tolerance of *S. uvarum* [8]. To gain insight into the function of the *MET4* gene, and validate the hypothesis that the *MET4* gene can regulate sulfite tolerance in *S. uvarum*, the *MET4* gene function in *S. uvarum* was mined by the construction of *MET4* overexpression strains, sulfite tolerance phenotype screening, polymerase chain reaction (PCR) analysis, fermentation weight loss analysis, transcriptome analysis, and RT-qPCR analysis.

## 2. Materials and Methods

*Saccharomycesuvarum* A9, pCAMBIA1301, and *E. coli* DH5α were stored at the Key Laboratory of Southwest Biodiversity Conservation, National Forest and Grassland Administration, Southwest Forestry University (Kunming, China). *MET4* was synthesized and constructed in Pgem-T Easy vector by Baiqi Biotechnology Co. (Wuhan, China) The sequence of the *MET4* gene is from the *S. uvarum* strain A9 [9] (access number: OL804291). Primers were synthesized by Shanghai Biotechnology Co., Ltd., Shanghai, China, and molecular reagents or kits were purchased from Shanghai Biotechnology Co.

### 2.1. Construction of the MET4 Gene Expression Vector

The small fragment of Pgem-T-*MET4*, cleaved by NcoI and BglII and purified according to [19], was ligated to the pCAMBIA1301 vector, which was also cleaved by these two enzymes. pCAMBIA1301-*MET4* was transferred into *E. coli* DH5α receptor cells by electrotransformation and then coated on LB (1% yeast extract, 1% tryptone, 2% agar, and 0.5% NaCl) plates containing 10 mg/mL Hygromycin (HYG) and incubated overnight at 37 °C. Larger transformed colonies were picked into 3 mL LB liquid medium (1% yeast extract, 1% tryptone, and 0.5% NaCl) containing 10 mg/mL HYG and incubated overnight at 37 °C. Cells were harvested, and plasmids were extracted and purified. The expression vector was then verified by Polymerase Chain Reaction (PCR) and sent to Shanghai Biotechnology for sequencing to test the success of the vector construction.

### 2.2. Genetic Transformation

After the preparation of *S*. *uvarum* receptor cells, the cells and recombinant plasmid pCAMBIA1301-*MET4* were mixed at a volume ratio of 10:1 in an electroporation cup and electroporated for 5 ms at 1500 V using an Eppendorf electroporator (Eppendorf, Hamburg, Germany). Eight hundred μL of YEP medium (1% yeast extract, 1% peptone, and 0.5% NaCl, pH 7.5) was added and incubated for 1 h at 28 °C. After incubation for 2 h in a 200 rpm shaker at 28 °C, the larger transformants were selected by incubating overnight on YPD (1% yeast extract, 2% peptone, 2% agar, and 2% glucose) plates containing 30 mg/L HYG and 30 mM sulfite.

### 2.3. Sulfite Tolerant Trait Typing

The strains were inoculated and grew on fresh YPD medium containing 5, 10, 20, 40, and 60 mM sodium sulfite and 80 mM succinate at pH 3.5. After 24 h, 48 h, 72 h, 96 h, 120 h, 144 h, and 168 h, the sulfite tolerance levels were recorded, according to colony growth measurements.

### 2.4. PCR Analysis

Deoxyribonucleic acid (DNA) samples were prepared according to the method of Nardi [20]. Ten candidate colonies were randomly selected from *MET4* transformants for PCR analysis. The PCR reaction mix (25 μL) consisted of 0.2 μL of 5 U/μL Taqase, 0.5 μL of 10 mM dNTP, 1 μL of 10 μM primer, 2 μL of DNA template, 2.5 μL of 10× PCR buffer (including mg^2+^), and 17.8 μL of dH_2_O. The primers used for PCR were HYG-F: 5′-TGCTGCTCCATACAAGCCAA-3′ and HYG-R: 5′-ACCGCAAGGAATCGGTCAAT-3. The PCR reactions were performed according to the following procedure: 95 °C pre-denaturation for 5 min; 35 cycles of denaturation at 95 °C for 30 s, annealing at 56 °C for 30 s, and extension at 72 °C for 60 s, with a final extension at 72 °C for 8 min.

### 2.5. Fermentation Weight Loss Analysis

The transgenic strain A9-*MET4* was fermented in 15 g of grape juice (grapes from Aziying, Panlong District, Kunming, China) containing 20 mM sodium sulfite and 80 mM succinic acid at pH 3.5, together with the starting strain A9 and EC1118. The Petri dishes containing the strains were initially weighed and their weight recorded as W0, weighed every 1 day for a total of 7 measurements as W1–7, and the weight loss every 1 day as W = Wn − Wn − 1. Based on these data, graphs were made in excel.

### 2.6. Ribonucleic Acid (RNA) Extraction and cDNA Synthesis

The yeast strains were collected by incubation in liquid YPD for 24 h, followed by treatment in a medium containing 20 mM sulfite for 10 min. Yeast cells were ground in liquid nitrogen and RNA was extracted using the QIAGEN kit (Qiagen China, Shanghai, China). RNA samples were then reverse transcribed into cDNA using the Reverse Transcription Kit (Takara, Dalian, China). These samples would be used for further RT-qPCR analysis and transcriptome analysis.

### 2.7. RT-qPCR Analysis

RT-qPCR analysis was performed using an ABI 7500 fluorescent qPCR instrument (Thermo Fisher Scientific, Carlsbad, CA, USA) according to the method described by Liu [9]. The primers are shown in Table 1. Actin-1 (*ACT1*) was used as the reference gene (in Table 1). Three replicates were set for each gene. Gene expression levels were calculated according to the method of Liu [8].

### 2.8. Transcriptome Analysis

RNA-Seq was performed by Nextomics Biosciences Co. Ltd. (Wuhan, China) using an Illumina HiSeqTM (Illumina, San Diego, CA, USA). RNA-Seq data were submitted to the National Center for Biotechnology Information (NCBI) (Bethesda, MD, USA) repository SRA (Accession no. PRJNA786265). Transcriptome assembly, sequence alignment, gene orthology determination, and gene set enrichment analysis were performed according to the method described by Bian [21]. Differential gene expression heat maps were plotted using Log2 (Fragments Per Kilobase of Exon Model per Million Mapped Fragments (FPKM) + 1) values with the R 3.0.2 software.

### 2.9. Data Analysis

Histograms were calculated and plotted using GraphPad Prism Software (v9.0), and one-way ANOVAs were performed by macros, with *p*-values and standard errors calculated using GraphPad Prism Software.

## 3. Results

### 3.1. Detection of Transgenic Strains

**Figure 1:**
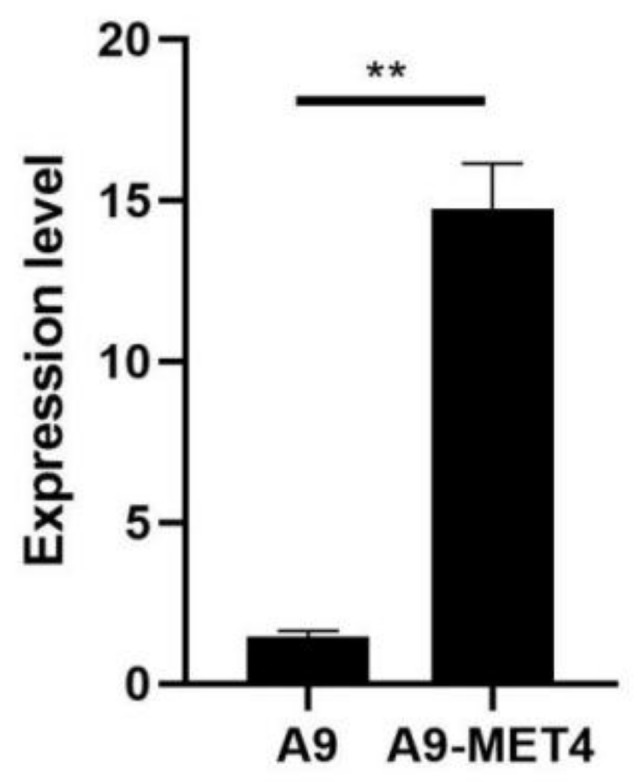
The expression level of the *MET4* gene in the *S. uvarum starting* strain A9 and its transformants. Enhanced expression or depression of the *MET4* gene was assessed using the 2^−ΔΔCT^ method to determine relative gene expression from RT-qPCR data with *ACT1* as a housekeeping gene. Values were means ± standard error (SE) of 2^−ΔΔCT^ (*n* = 3). ** p *<* 0.01.

Transformants grown on sulfite-containing media were analyzed by PCR, and the results showed that all ten transformants picked were positive. Three of the larger transformants grown in sulfite-tolerant medium were picked for RT-qPCR analysis, which showed that their relative expression was 10.11-fold higher than the mean value of the starting strain (see Figure 1), with the difference reaching significance (*p*-value < 0.01).

Comparison with transformants transfected with the *SSU1* gene and the *FZF1* gene indicates that the transfection of this gene can enhance sulfite tolerance in grape juice yeast, but to a lesser extent than the overexpression transformants of the two genes mentioned above (See Table 2) [8,9].

**Figure 2:**
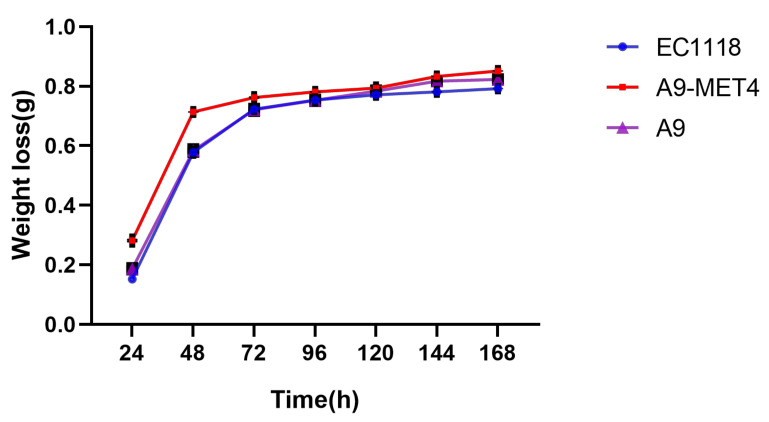
Weight loss curve of the transformant. Three replicates were set for each strain, each point in the figure was the mean value of three values. Values were means ± SE.

During the first three days of fermentation, the weight loss of the *MET4* transformants was significantly higher than that of the starting strain. As the fermentation progressed, the weight loss of the *MET4* transformants was still higher than that of the starting strain, but the value of the difference decreased (see Figure 2). It indicates that the *MET4* transformants were significantly more capable of fermenting in grape juice containing 20 mM sodium sulfite than the starting strain in the early stages of fermentation. As time progressed, the weight loss between the *MET4* transformants and the starting strain fermentate gradually drew closer as the sugars were gradually consumed, but the weight loss of the *MET4* transformants fermentate was still higher than that of the starting strain. It indicates that the fermentability of *MET4* transformants in sulfite-containing grape juice was also higher than that of the starting strain in the later stages of fermentation.

### 3.2. Transcriptome Differential Gene Analysis

Transcriptome sequencing yielded over 250 million high-quality reads, with over 4 million high-quality gene data obtained per replicate. Only 1.1% of FPKM values were between 0–1 in A9, and 2.5% of FPKM values were between 0–1 in A9-*MET4*. In the A9 strain, 86.1% of FPKM values were above 10, and 75.8% of FPKM values were above 10 in the A9-*MET4* strain (Table 3). These results demonstrate the high quality of the reads obtained.

**Figure 3:**
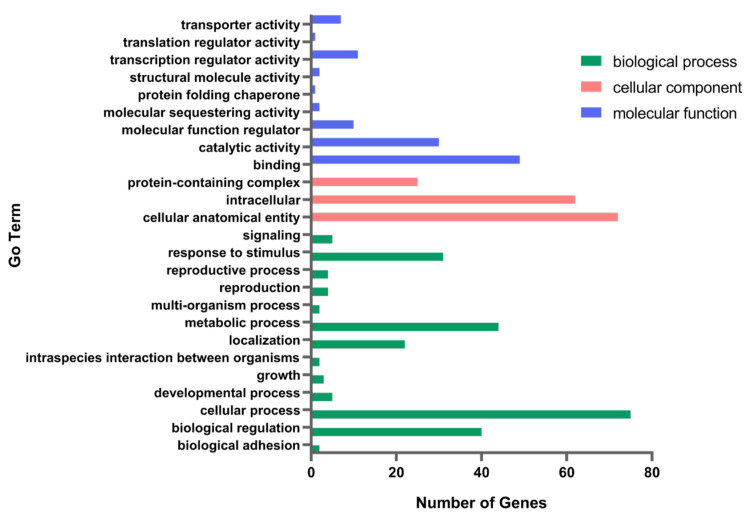
Pathway enrichment of differentially expressed genes between A9-*MET4* and the starting strain A9 with Gene Ontology (GO) interpretation.

The transcriptome profiles of A9-*MET4* and the starting strain A9 were compared, and a total of 92 differential genes were obtained, with 90 upregulated genes and two downregulated ones. These differential genes were annotated to the three major functional classes, such as molecular function, cellular component, and biological process (Figure 3). The differentially expressed cellular-component-related genes are mainly involved in protein-containing complexes, cellular anatomical entities, and intracellular proteins. Molecular functional genes include genes related to binding, catalytic activity, transcriptional activity, transport activity, and protein folding chaperones, while biological-process-related genes are related to cellular processes, metabolic processes, bioregulation, and response to stimuli. The expression of genes related to fermentation-stress response, protein folding, and transcriptional regulation of RNA polymerase II was upregulated (see Table 4), e.g., *HOM3*, *NRG1*, and *VID24*. The methylenetetrahydrofolate dehydrogenase gene *MIS1*, which is related to NADPH synthesis, is downregulated.

**Figure 4:**
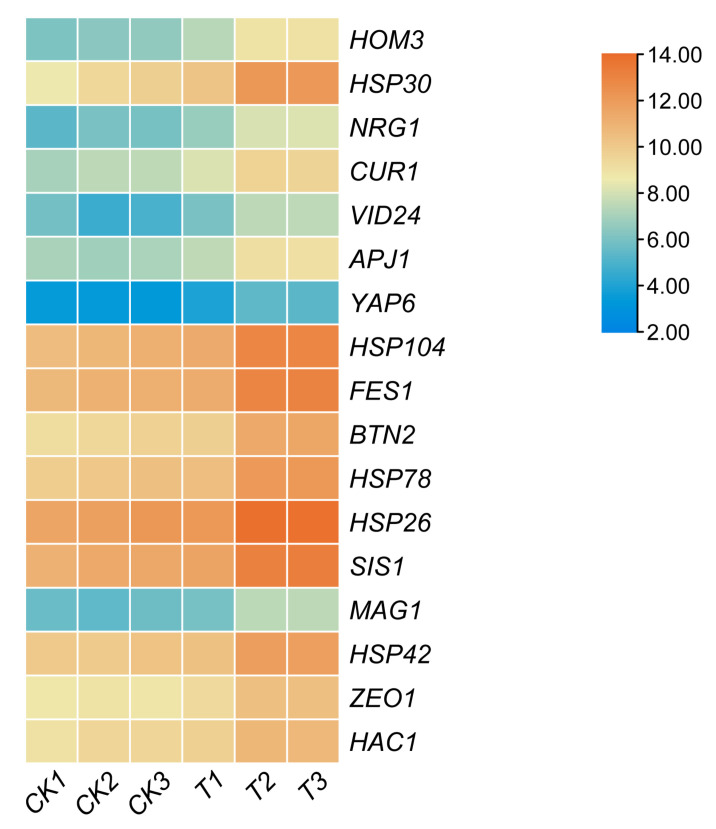
Heat map of the expression levels of differentially expressed, stress-related genes. Color represents expression change. CK1, CK2, and CK3, control; T1, T2, and T3, A9-*MET4,* treated in a sulfite-containing medium for 10 min.

An expression heat map analysis was performed for genes involved in the stress response (Figure 4). The eight most differentially expressed genes compared to the starting strain A9 were *HOM3*, *HSP30*, *NRG1*, *VID24*, *APJ1*, *YAP6*, *Hsp104*, and *FES1*. The differential expression of these genes may be associated with increased sulfite tolerance.

**Figure 5:**
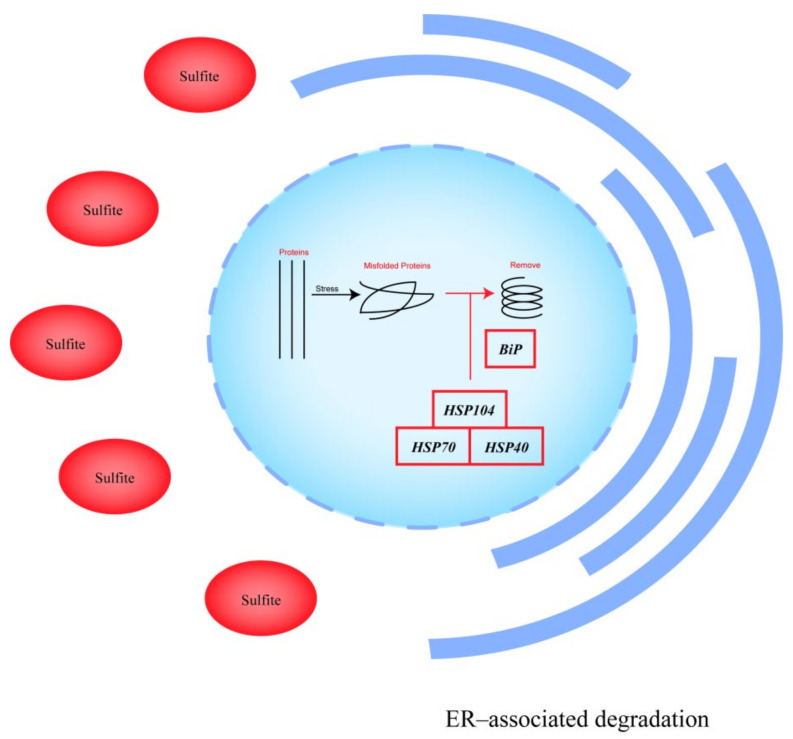
Map of protein processing pathways in the endoplasmic reticulum of A9-*MET4* in a sulfite environment. The main upregulated genes in the figure are: (a) *HSP104*, which regulates the expression of the nucleotide-exchange factor (*NEF*), with an expression level Log2 value of 1.50; (b) *KAR2*, which regulates the expression of *BiP* (Binding immunoglobulin protein), with a Log2 value of 1.29; (c) *SSA4*, which regulates the expression of the *HSP70* family, with a Log2 value of 1.43; (d) *APJ1*, which regulates the expression of the *HSP40* family, with a Log2 value of 1.61. *HSP104* combined with *HSP70* and *HSP40* activates denatured protein refolding under stress conditions [26]. The figure is adapted from the KEGG pathway.

To identify the major pathways affecting sulfite tolerance in *S. uvarum*, differential expression genes (DEGs) were analyzed for enrichment in the KEGG (Kyoto Encyclopedia of Genes and Genomes) pathway website [22]. Among these, protein processing in the endoplasmic reticulum was the most significantly enriched pathway for differential genes, with a *p*-value = 0.002 < 0.05 (see Figure 5). Most upregulated genes were enriched in the endoplasmic reticulum-associated degradation (ERAD) response, e.g., *Hsp104*, *HSP*78, etc.; the molecular chaperone binding protein (BiP) was also enriched with an uregulated gene, and BIP induces the ERAD response [23]. Isolation of misfolded proteins into insoluble aggregates under proteotoxic stress conditions is a way for cells to attempt to maintain function [24], and the ERAD response can remove misfolded proteins in response to stress [25]. This pathway suggests that *MET4* overexpression in *S. uvarum* transformants enhance their sulfite adaptation by activating the ERAD response.

In the cysteine and methionine metabolic pathways, the gene encoding aspartate kinase (*HOM3*, EC: 2.7.2.4) is differentially expressed in a sulfite environment. Aspartate kinase is the enzyme of the first step in methionine biosynthesis [27]. In this study, *HOM3* was the most differentially expressed gene, which may indicate that overexpression of *MET4* in *S. uvarum* regulates methionine synthesis by increasing *HOM3* expression, thereby ultimately enhancing its sulfite adaptation.

### 3.3. RT-qPCR Validation

**Figure 6:**
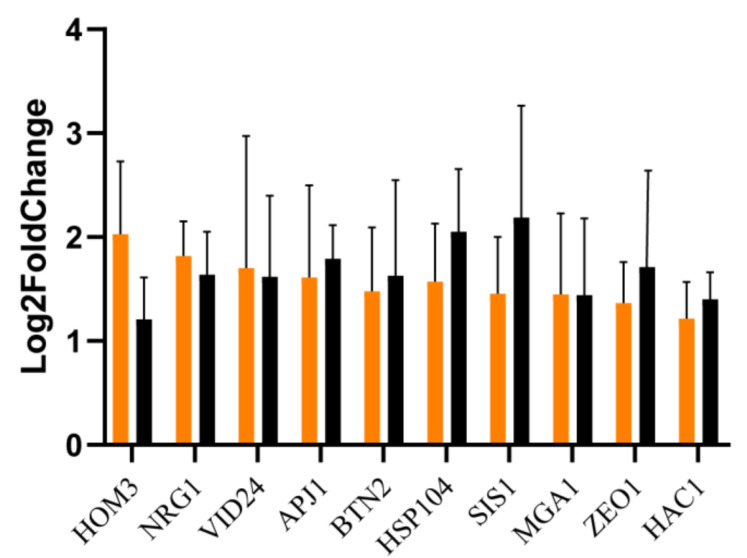
Comparison of transcriptome and RT-qPCR analysis results. Orange, transcriptome analysis; Black, RT-qPCR analysis. The standard deviation values of the treatments (error bars) are treated with log10 because they are too large after dividing by the control values; the rest are log2 values. Each gene was replicated three times.

The results of the RT-qPCR analysis confirmed that the ten genes selected in the transcriptome analysis were indeed upregulated in the *MET4* transformants compared to the starting strain, although the correlation coefficient (R^2^ = 0.0659) between these two sets of data was small. Both the transformants and the starting strain were treated with the same method and for the same duration, but the expression of genes involved in sulfite depletion (e.g., *HOM3*) and stress-related genes (e.g., *Hsp104*) were significantly upregulated in the transformants (see Figure 6). It suggests that the overexpression of the *MET4* gene promotes the expression of genes involved in sulfite depletion and stress-related genes, which in turn leads to enhanced sulfite tolerance in *S. uvarum*.

## 4. Discussion

In winemaking, the sulfite assimilation pathway in *S. cerevisiae* has been revealed to be controlled by five transcriptional regulators, including three DNA-binding proteins (Met31p, Met32p, and Cbf1p), an activator (MET4p), a cofactor (Met28p), and a combination of ubiquitin ligase subunits (Met30p). Of these, *MET4*p is the only transcriptional activator in the sulfite assimilation pathway [28]. In a strain of *S. uvarum* isolated from wine that exhibits high sulfite tolerance, the sulfite tolerance is regulated through the *FZF1* gene, not *SSU1* [9]. In a previous study, we compared the transcriptional profiles of *FZF1*-overexpressing and *FZF1*-silenced *S. uvarum* transformants and found that the *FZF1* gene regulates *S. uvarum* sulfite tolerance by modulating *MET4* gene expression levels [9]. The different expression levels of *MET4* may be one of the important reasons for the different sulfite tolerance observed in the *MET4* transformants and the starting strain. The *MET4* transformants were sulfite tolerant up to 40 mM, which was 20 mM higher than the 20 mM of the starting strain A9, but 60 mM and 40 mM lower than the transformants of the *FZF1* and *SSU1* genes, respectively, suggesting that the *MET4* gene could play a smaller role in sulfite tolerance in *S. uvarum* strains than the *FZF1* and *SSU1* genes.

After a comparison in the SGD database (Data updated on 1 February 2022) [29], we found that the genes in the expression profile of the *MET4* overexpression *S. uvarum* transformants did not have any overlapping parts with the *S. cerevisiae MET4* reciprocal gene [30,31], indicating that the *MET4* gene of the *S. uvarum* A9 strain is functionally distinct from the *MET4* gene of *S. cerevisiae*. Both the *MET4* gene of *S. uvarum* and *S. cerevisiae* contain a basic leucine zip structural domain (bZip) at the end, but about 29.57% of the DNA sequence of *S. uvarum MET4* is different from that of *S. cerevisiae MET4* (NM_001182941.3, *S. cerevisiae* S288C) and the encoded amino acids differ by about 33.24%; these differences may be the main reason for the change in gene function of *MET4*.

Fermentation of grape juice in the presence of sulfite exposes the yeast to a cascade of stresses including osmotic pressure, hypoxia, nitrogen depletion, and increased ethanol concentration. Fermentation-stress-response genes exhibit sustained and significantly induced expression in response to stress conditions during fermentation. In this study, fermentation-stress-response and heat-stress-protein-encoding genes accounted for approximately 40% of the differentially upregulated expressed genes in the expression profile of the *MET4* overexpression *S. uvarum* transformants. The significant upregulation of these genes facilitated the sustained fermentation of *S. uvarum* in a sulfite environment. In this study, RNA-polymerase-II-related genes were upregulated, and the methylenetetrahydrofolate dehydrogenase gene MIS1, associated with NADPH synthesis, was downregulated in the *MET4* overexpression *S. uvarum* transformants compared to the starting strain. The reason for this might be due to the positive regulatory function of the *MET4* gene on RNA polymerase II on transcription, and the toxic effect of sulfite on enzymes associated with NADPH production or utilization [32,33].

In the sulfate assimilation pathway, sulfite is reduced by sulfite reductase and eventually synthesized into sulfur-containing compounds. It has been shown that *HOM3* is involved in regulating the synthesis of the sulfur-containing compound methionine [34], and can consume a portion of the sulfite. In the present study, the overexpression of the *MET4* gene in *S. uvarum* upregulated the expression of the *HOM3* gene. We suggested that upregulating the expression of the *HOM3* gene depleted more of the sulfite in the medium, causing the concentration of sulfite in the environment to decrease, which in turn allowed the transformants to survive or ferment in succession with higher levels of sulfite, i.e., to be more tolerant of sulfite. Therefore, the upregulation of the expression of *HOM3* might be one of the key reasons for the increased sulfite tolerance in *MET4 S. uvarum* transformants. Some populations are allergic to sulfite, and common symptoms in sulfite-allergic people after exposure to sulfite include nasal congestion, headache, breathing difficulties, nausea, dizziness, and abdominal pain. The *MET4 S. uvarum* transformants obtained in this study could deplete the sulfite in its fermentation environment, which in turn would further reduce the adverse effects of its fermentation products on sulfite-allergic people.

Mechanisms of sulfite tolerance in *S. cerevisiae* have been uncovered, including (A) the mediation of sulfite efflux via *SSU1*p and *FZF1*p, (B) the synthesis of non-toxic compounds with acetaldehyde, and (C) the reduction of sulfite in vivo [11]. Of these, sulfate reduction is mainly via the reduction to sulfur-containing compounds by sulfite reductase in the sulfate assimilation pathway [10]. We suggested that in this study, *MET4* overexpression transformants may also deplete the sulfite in the culture environment through sulfate assimilation, thereby reducing the sulfite concentration in the environment (i.e., medium) and achieving higher sulfite concentration tolerance. Interestingly, we found that in *S. uvarum*, the upregulation of *SSU1* gene expression was accompanied by increased expression of the *HOM3* gene [8], but that the upregulation of *FZF1* expression was not accompanied by a corresponding increase in *HOM3* gene expression [9].

It has been suggested that the *Hsp104* gene is associated with ethanol tolerance [24]. It has been further shown that other members of the HSP gene family are also upregulated in other adverse environments [35]. The expression of *Hsp104* is elevated in *FZF1* overexpressing *S. uvarum* strains [9] and upregulated in *MET4* overexpressing *S. uvarum* strains. The elevated expression of the *Hsp104* gene in the present study suggested that this gene may be associated with the ability of transformants to tolerate higher concentrations of sulfite.

## 5. Conclusions

(1) We suggest that a mechanism of sulfite tolerance exists in *S. uvarum*, i.e., tolerance to higher sulfite concentrations is achieved by depleting the sulfite in the culture environment. *S. uvarum* may be able to tolerate higher concentrations of sulfite by continuously growing and dividing, and by upregulating the expression of genes, such as *HOM3*, which consume part of the sulfite in the culture medium or fermentation broth.
(2) *S. uvarum* may be able to tolerate higher concentrations of sulfite by increasing the expression of *MET4*, and thus the stress-related genes. The hypothesis that the ‘*MET4* gene can regulate sulfite tolerance in *S. uvarum*’ is proved.
(3) Overexpression of the *MET4* gene resulted in a significant increase in sulfite tolerance, an upregulation of fermentation-stress-related gene expression, and a significant change in the transcriptome profile of the strain. The transformants could broaden the application of *S. uvarum* in the winemaking industry.

## Figures and Tables

**Table 1:** Primers used for RT-qPCR and PCR analysis.

Gene	Sequence (5′–3′)	Product Length
*HOM3*	TAA ATG GTG TCG GTC GTG	235
	TTG GCT CTG ATA ACT TGC T	
*NRG1*	ATT TCG GCG TTT GAT AGA	304
	CAT TCA GTT GGG ATA GCG	
*VID24*	GTA GAC AGT TTG GCG GAG AA	155
	CGG TCA ACG AGA CGG AAT	
*APJ1*	CTT TGA CAC GGG AGG AGT	146
	GGA AGT TGG GCG TAG AGC	
*BTN2*	GTT GAA CCA TTC TAT CCC TC	218
	GAT TCC TTC TTG GCT TTT	
*Hsp104*	AAG AAT TGA CTC CCG TGG TG	193
	ACC TGG CTC ACC AAT CAA AC	
*SIS1*	GCC AAC AGG GGA TAC TGA AA	227
	TGA AAG CGT CTT CAT TGC TG	
*MGA1*	TCT GAA ACC GTA TGA CCC	273
	TTA CCA TCT TTG CCC ACA	
*ZEO1*	AGC TGG ATG AAA CTA AGG A	174
	TGG TGG TGA CTT CGG TCT	
*HAC1*	CAA GAC GGA GAA CAT ACA AGA	177
	ATC GTA ATC ACG GCT GGA	
*MET4*	TCG CAG TAT GAC CAA TCC AA	163
	CAG CCG TGC TTA CAG GAA AT	
*ACT1*	AGC GCA ATC CAA GAG AGG TA	153
	GCT TCG GTC AAA AGA ACA GG	

Note: *HOM3*, HOMoserine requiring gene 3; *NRG1*, Negative regulator of glucose-repressed gene 1; *VID24*, Vacuolar import and degradation gene 24; *APJ1*, Anti-prion DnaJ gene 1; *BTN2*, BaTteN disease gene 2; *Hsp104*, Heat shock protein encoding gene 104; *SIS1*, SIt4 suppressor gene 1; *MGA1*, *M*egaloblastic anaemia gene 1; *ZEO1*, Zeocin-1 gene; *HAC1*, Histone acetyltransferase gene 1; *MET4*, Methionine synthase gene 4; *ACT1*, Actin-1 gene.

**Table 2:** The genotype and sulfite resistance ability of *MET4* transgenic strain and its starting stain.

Strain	HYG	Sodium Sulfite (mM)				
		5	10	20	40	60
A9	−	+	+	+	−	−
A9-MET4	+	+	+	+	+	−

**Table 3:** Differential expression analysis of different stains.

	Sample	CK1	CK2	CK3	T1	T2	T3
FPKM							
0–1		1.2%	1.1%	1.2%	1.1%	2.5%	2.5%
1–10		12.7%	13.5%	13.1%	12.1%	21.6%	20.9%
≥10		86.1%	85.4%	85.7%	86.9%	75.9%	76.6%

**Table 4:** The gene expression levels of A9-*MET4* compared to the starting strain A9.

KEGG Category	Gene	Log2	Decribution
Amino sugar and nucleotide sugar metabolism	*CHS2* (CHitin Synthase 2)	1.54	Chitin synthase
	*LPX1* (Lipase of PeroXisomes 1)	1.24	Chitinase
Autophagy-yeast	*LST8* * (Lethal with Sec Thirteen 8)	1.62	Target of rapamycin complex subunit LST8
Endocytosis	*SSA4* * (Stress-Seventy subfamily A 4)	1.43	Heat shock 70 kDa protein
	*ENT2* (Epsin N-Terminal homology 2)	1.40	Epsin-like protein required for endocytosis and actin patch assembly
RNA transport	*BTN2* (BaTteN disease 2)	1.48	Translation initiation factor
	*DI49_2214* (Unannotated)	1.23	Polyadenylate-binding protein
Roibosome biogenesis in eukaryotes	*NOP4* (NucleOlar Protein 4)	1.29	Nucleolar protein 4
Spliceosome	*SLU7* (Synergistic Lethal with U5 snRNA 7)	1.37	Pre-mRNA-processing factor
	*SSA4* * (Stress-Seventy subfamily A 4)	1.43	Heat shock 70 kDa protein
Mitogen-Activated Protein Kinase (MAPK) signaling pathway-yeast	*TEC1* (Transposon Enhancement Control 1)	1.41	Transcriptional enhancer factor
	*DI49_4478* (Unannotated)	0.64	Cytokinesis protein
Non-homologous end-joining	*DNL4* (DNA Ligase 4)	0.86	DNA ligase 4
ATP-binding cassette (ABC) transporters	*PDR12* (Pleiotropic Drug Resistance 12)	0.87	ATP-binding cassette
Vitamin B6 metabolism	*SNO1* (SNZ proximal Open reading frame 1	1.20	5′-Phosphate synthase pdxT subunit
Sphingolipid metabolism	*LCB5* (Long-Chain Base 5)	1.43	Sphingosine kinase
Mannose type O-glycan biosynthesis	*ZEO1* * (ZEOcin resistance 1)	1.36	Dolichyl-phosphate-mannose-protein mannosyltransferase
Glycosylphosphatidylinositol (GPI)-anchor biosynthesis	*MCD4* (Morphogenesis Checkpoint Dependent 4)	1.27	Phosphatidylinositol glycan
	*GPI12* (GlycosylPhosphatidylInositol anchor biosynthesis 12)	1.54	*N*-acetylglucosaminylphosphatidylinositol deacetylase
Protein processing in the endoplasmic reticulum	*APJ1* * (Anti-Prion DnaJ gene 1)	1.61	DnaJ homolog subfamily A member 2
	*FES1* * (Factor Exchange for Ssa1p 1)	1.50	*Hsp70*-interacting protein
	*HSP26* * (Heat Shock Protein 26)	1.47	HSP20 family protein
	*SSA4* * (Stress-Seventy subfamily A 4)	1.43	Heat shock 70 kDa protein
	*SSE1* * (Stress Seventy subfamily E 1)	1.41	Heat shock protein 110 kDa
	*HSP42* * (Heat Shock Protein 42)	1.37	HSP20 family protein
	*KAR2* * (KARyogamy 2)	1.29	Heat shock 70 kDa protein 5
	*DER1* * (Degradation in the Endoplasmic Reticulum 1)	1.26	Derlin-2/3
	*MPD1* * (Multicopy suppressor of PDI1 deletion 1)	1.08	Protein disulfide-isomerase A6
Meiosis-yeast	*RPI1* * (Ras-cAMP Pathway Inhibitor 1)	1.33	Mediator of RNA polymerase II transcription subunit
	*PHD1* (PseudoHyphal Determinant 1)	1.29	Enhanced filamentous growth protein 1
Cysteine and methionine metabolism	*HOM3* * (HOMoserine requiring gene 3)	2.03	Aspartate kinase
Lysine degradation	*DI49_2072* (Unannotated)	1.31	Lysine *N*-acetyltransferase
	*SET2* (SET domain-containing 2)	1.34	Histone-lysine *N*-methyltransferase SETD2
Longevity regulating pathway-multiple species	*Hsp104* * (Heat Shock Protein 104)	1.57	ATP-dependent Clp protease ATP-binding subunit ClpB
	*HSP78* * (Heat Shock Protein 78)	1.48	ATP-dependent Clp protease ATP-binding subunit ClpB
	*SSA4* * (Stress-Seventy subfamily A 4)	1.43	Heat shock 70 kDa protein
	*RPI1* * (Ras-cAMP pathway inhibitor 1)	1.33	Mediator of RNA polymerase II transcription subunit

Note: *, Fermentation-stress-related gene.

## Data Availability

All data generated or analyzed during this study are included in this published article. RNA-Seq data were presented at the Genome Sequence Archive of the Beijing Institute of Genomics (BIG) Data Center (accession number CRA001986).

## References

[1] Zhang H.Y., Lee S.A., Bradbury J.E., Warren R.N., Sheth H., Hooks D.O., Richards K.D., Gardner R.C. (2010). Yeasts isolated from New Zealand vineyards and wineries. Aust. J. Grape Wine Res..

[2] Zhang H., Richards K.D., Wilson S., Lee S.A., Sheehan H., Roncoroni M., Gardner R.C. (2015). Genetic characterization of strains of *Saccharomyces uvarum* from New Zealand wineries. Food Microbiol..

[3] Nadai C., Treu L., Campanaro S., Giacomini A., Corich V. (2015). Different mechanisms of resistance modulate sulfite tolerance in wine yeasts. Appl. Microbiol. Biotechnol..

[4] Yang Y. (2007). Formation and characteristics of wine bouquet produced by wine yeasts. Microbiology.

[5] Liu X.Z., Zhang Z.M., Zhang H.Y. (2017). Cross breeding and hybrid identification of sulphite-tolerant hybrids of *Saccharomyces uvarum*. S. Afr. J. Enol. Vitic..

[6] Vargas-Maya N.I., González-Hernández G.A., Padilla-Guerrero I.E., Torres-Guzmán J.C. (2017). Overexpression of *smORF YNR034W-A/EGO4* in *Saccharomyces cerevisiae* increases the fermentative efficiency of *Agave tequilana* Weber must. J. Ind. Microbiol. Biotechnol..

[7] Bergman A., Vitay D., Hellgren J., Chen Y., Nielsen J., Siewers V. (2019). Effects of overexpression of *STB5* in *Saccharomyces cerevisiae* on fatty acid biosynthesis, physiology and transcriptome. FEMS Yeast Res..

[8] Liu X.Z., Sang M., Zhang X.A., Zhang T.K., Zhang H.Y., He X., Li S.X., Sun X.D., Zhang Z.M. (2017). Enhancing expression of *SSU1* genes in *Saccharomyces uvarum* leads to an increase in sulfite tolerance and a transcriptome profile change. FEMS Yeast Res..

[9] Liu X., Liu X., Zhang Z., Sang M., Sun X.D., He C.Z., Xin P.Y., Zhang H.Y. (2018). Functional *a*nalysis of the *FZF1*
*g*enes of *Saccharomyces uvarum*. Front. Microbiol..

[10] Hansen J., Johannesen P.F. (2000). Cysteine is essential for transcriptional regulation of the sulfur assimilation genes in *Saccharomyces cerevisiae*. Mol. Gen. Genet..

[11] Thomas D., Surdinkerjan Y. (1997). Metabolism of sulfur amino acids in *Saccharomyces cerevisiae*. Microbiol. Mol. Biol. Rev..

[12] Kuras L., Thomas D. (1995). Functional analysis of *MET4*, a yeast transcriptional activator responsive to S-adenosylmethionine. Mol. Cell. Biol..

[13] He X., Zhang X.A., Liu X.Z., Li S.X., Zhang H.Y. (2015). Cloning and sequence analysis of the *FZF1* gene concerning sulfur tolerance from *Saccharomyces bayanus*. Sci. Technol. Food Ind..

[14] Thomas D., Jacquemin I., Surdin-Kerjan Y. (1992). *MET4*, a leucine zipper protein, and centromere-binding factor 1 are both required for transcriptional activation of sulfur metabolism in *Saccharomyces cerevisiae*. Mol. Cell. Biol..

[15] Kuras L., Cherest H., Surdin-Kerjan Y., Thomas D. (1996). A heteromeric complex containing the centromere binding factor 1 and two basic leucine zipper factors, *MET4* and *Met28*, mediates the transcription activation of yeast sulfur metabolism. EMBO J..

[16] Su N.Y., Ouni I., Papagiannis C.V., Kaiser P. (2008). A dominant suppressor mutation of the *Met30* cell cycle defect suggests regulation of the *Saccharomyces cerevisiae MET4*-*Cbf1 t*ranscription complex by *Met32*. J. Biol. Chem..

[17] Kuras L., Barbey R., Thomas D. (1997). Assembly of a *bZIP-bHLH* transcription activation complex: Formation of the yeast *Cbf1*-*MET4*-*Met28* complex is regulated through *Met28* stimulation of *Cbf1* DNA binding. EMBO J..

[18] Blaiseau P.L., Thomas D. (2014). Multiple transcriptional activation complexes tether the yeast activator *MET4* to DNA. EMBO J..

[19] Sambrook J., Fritsch E.F., Maniatis T. (1989). Molecular Cloning: A Laboratory Manual, New York: Cold Spring Harbor Laboratory Press. BioScience.

[20] Nardi T., Corich V., Giacomini A., Blondin B. (2010). A sulphite-inducible form of the sulphite efflux gene *SSU1* in a *Saccharomyces cerevisiae* wine yeast. Microbiology.

[21] Bian W., Liu X., Zhang Z., Zhang H. (2020). Transcriptome analysis of diploid and triploid *Populus tomentosa*. Peer J..

[22] Kanehisa M., Goto S. (2000). KEGG: Kyoto encyclopedia of genes and genomes. Nucleic Acids Res..

[23] Wakasa Y., Yasuda H., Oono Y., Kawakatsu T., Hirose S., Takahashi H., Hayashi S., Yang L., Takaiwa F. (2015). Expression of ER quality control-related genes in response to changes in *BiP1* levels in developing rice endosperm. Plant J. Cell Mol. Biol..

[24] Sathyanarayanan U., Musa M., Dib P.B., Raimundo N., Milosevic I., Krisko A. (2020). ATP hydrolysis by yeast *Hsp104* determines protein aggregate dissolution and size in vivo. Nat. Commun..

[25] Shi J., Hu X., Guo Y., Wang L., Ji J., Li J., Zhang Z.R. (2019). A technique for delineating the unfolding requirements for substrate entry into retrotranslocons during endoplasmic reticulum-associated degradation. J. Biol. Chem..

[26] Glover J.R., Lindquist S. (1998). *Hsp104*, *Hsp70*, and *Hsp40*: A novel chaperone system that rescues previously aggregated proteins. Cell.

[27] Rafalski J.A., Falco S.C. (1988). Structure of the yeast *HOM3* gene which encodes aspartokinase. J. Biol. Chem..

[28] Lee T.A., Jorgensen P., Bognar A.L., Peyraud C., Thomas D., Tyers M. (2010). Dissection of combinatorial control by the *MET4* transcriptional complex. Mol. Biol. Cell.

[29] Park H., Bakalinsky A.T. (2000). *SSU1* mediates sulphite efflux in *Saccharomyces cerevisiae*. Yeast.

[30] Thorsen M., Lagniel G., Kristiansson E., Junot C., Nerman O., Labarre J., Tamas M.J. (2007). Quantitative transcriptome, proteome, and sulfur metabolite profiling of the *Saccharomyces cerevisiae* response to arsenite. Physiol. Genom..

[31] Youn J.Y., Friesen H., Nguyen Ba A.N., Liang W., Messier V., Cox M.J., Moses A.M., Andrews B. (2017). Functional analysis of kinases and transcription factors in *Saccharomyces cerevisiae* using an integrated overexpression library. G3 Genes|Genomes|Genetics.

[32] Kobayashi K., Yoshimoto A. (1982). Studies on yeast sulfite reductase. IV. Structure and steady-state kinetics. Biochim. Biophys. Acta.

[33] McIsaac R.S., Petti A.A., Bussemaker H.J., Bussemaker D. (2012). Perturbation-based analysis and modeling of combinatorial regulation in the yeast sulfur assimilation pathway. Mol. Biol. Cell.

[34] Arévalo-Rodríguez M., Calderón I.L., Holmberg S. (2010). Mutations that cause threonine sensitivity identify catalytic and regulatory regions of the aspartate kinase of *Saccharomyces cerevisiae*. Yeast.

[35] Chen J., Gao T., Wan S., Zhang Y.H., Yang J.K., Yu Y.B., Wang W.D. (2018). Genome-wide identification, classification and expression analysis of the *HSP* gene superfamily in tea plant (*Camellia sinensis*). Int. J. Mol. Sci..

